# Edge Detection via Fusion Difference Convolution

**DOI:** 10.3390/s23156883

**Published:** 2023-08-03

**Authors:** Zhenyu Yin, Zisong Wang, Chao Fan, Xiaohui Wang, Tong Qiu

**Affiliations:** 1 Shenyang Institute of Computing Technology, Chinese Academy of Sciences, Shenyang 110168, China; wangzisong21@mails.ucas.ac.cn (Z.W.); fanchao18@mails.ucas.ac.cn (C.F.); wangxiaohui201@mails.ucas.ac.cn (X.W.); Z2021361@stu.syuct.edu.cn (T.Q.); 2 University of Chinese Academy of Sciences, Beijing 100049, China; 3 School of Computer Science and Technology, Shenyang University of Chemical Technology, Shenyang 110142, China

**Keywords:** edge detection, deep learning, contour detection, boundary detection, segmentation

## Abstract

Edge detection is a crucial step in many computer vision tasks, and in recent years, models based on deep convolutional neural networks (CNNs) have achieved human-level performance in edge detection. However, we have observed that CNN-based methods rely on pre-trained backbone networks and generate edge images with unwanted background details. We propose four new fusion difference convolution (FDC) structures that integrate traditional gradient operators into modern CNNs. At the same time, we have also added a channel spatial attention module (CSAM) and an up-sampling module (US). These structures allow the model to better recognize the semantic and edge information in the images. Our model is trained from scratch on the BIPED dataset without any pre-trained weights and achieves promising results. Moreover, it generalizes well to other datasets without fine-tuning.

## 1. Introduction

Edge detection, as a traditional computer vision task, aims to identify prominent changes in brightness or discontinuous regions in an image, making it an important research area in feature extraction. It provides fundamental information for many advanced visual tasks, such as image segmentation [1,2], contour extraction [3], object detection [4,5,6], and 2D object recognition [7]. With the advancement of deep learning, new domains, such as medical image analysis and remote sensing, have emerged, which often require an edge detection system. Consequently, the edge detection problem has been reexamined to address challenges such as complex backgrounds, inconsistent annotation labels, and speed improvements.

The primary objective of edge detection is to identify points in an image with significant brightness variations, which are closely related to the semantic clues of the image. Obtaining appropriate low-level or high-level image features through suitable methods is crucial. Early traditional methods can be categorized into two main types: The first type involves utilizing first-order or second-order operators, which rely on the gradient or derivative information of the image to extract edge information. Commonly used operators include Robert, Prewitt, Sobel [8], Canny [9], and generalized Laplacian of Gaussian (gLOG) [10]. The second type involves the manual design of features related to natural boundaries, such as brightness, color, and texture, which are then used to train classifiers. Examples of such methods include Pb and gPb [11]. These traditional methods remain effective in scenarios in which high accuracy is not required. For instance, the traditional approach of using pixel grayscale gradients is employed to determine the edges of the images [5].

In the era of deep learning, with the remarkable success of deep convolutional networks in image classification and other domains, convolutional neural networks (CNNs) have been widely applied to edge or contour detection in images. Based on the structure of these networks, they can be categorized into three major types. Non-end-to-end deep learning models are one type; N4-Fields [12] is a commonly used method. Another type, end-to-end multi-scale deep learning models, is represented by the holistically-nested edge detection (HED) [13]. Numerous studies have made improvements to this model, such as RDS [14], CASENet [15], and DexiNed [16]. A third type, encoder–decoder architectures, includes CEDN [17] and EDTER [18] as common examples. In addition to these mainstream methods, there are several important research directions, including lightweight models such as Pidinet [19], unsupervised learning models [20], and multi-task learning models [21]. These network structures are mostly based on multi-level feature extraction and fusion, along with well-designed training modes using appropriate loss functions, resulting in promising results.

However, in recent years, few network structures for edge detection have integrated traditional gradient operators into modern convolutional neural networks. In some studies, such as GSCNN [22], the Canny operator is introduced to reduce the loss of image resolution caused by network depth and pooling operations. Pidinet [19] proposes a new convolutional difference structure (PDC) to achieve lightweight and efficient edge detection models. However, the aforementioned deep learning models have the following limitations. The edges generated by these models are typically multi-pixel edges. Although there are methods to generate thin edges by modifying the loss function [23], they may also extract unnecessarily detailed edge information from the background, resulting in suboptimal results. Therefore, most of these models require non-maximum suppression (NMS) to be applied during evaluation. These models are susceptible to noise and texture, leading to the highlighting of more unnecessary texture information in the final results.

We have noticed that most previous methods were trained and tested on commonly used datasets for boundary detection or semantic segmentation, such as BSDS [24], MDBD [25], NYUD [26], and PASCAL [27]. However, these datasets suffer from missing or incorrect annotations of edges, and some of them are not specifically designed for edge detection.

**Figure 1:**
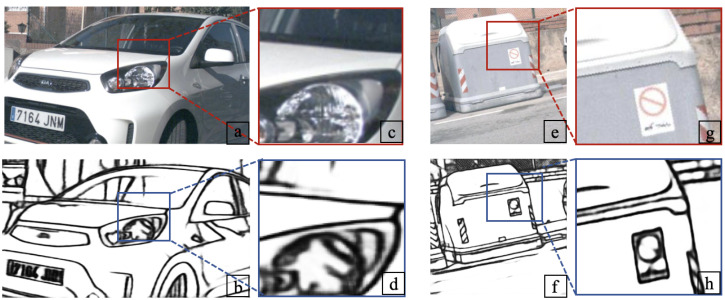
Examples of our edge detection. Our method extracts clear boundaries and edges by exploiting both global and local cues. (**a**,**e**): Two randomly selected images from the test set of the BIPED dataset; (**b**,**f**): The edge images obtained through the method presented in our paper; (**c**,**d**,**g**,**h**): Enlarged detail images of edge images.

To address the aforementioned issues, this paper proposes modifications to the Xception model and trains it from scratch without relying on pre-trained model weights. The proposed approach achieves promising results (ODS 0.857) on the BIPED dataset [16] and demonstrates good generalization to other datasets without fine-tuning. Figure 1 shows two examples of edge detection. The main contributions of this paper are as follows:

(1) Improvement of the original PDC structure by adding a vanilla convolutional layer. This enhancement allows for better extraction of both semantic and edge information from images, reducing the impact of texture information and noise while maintaining similar computational cost and memory usage to the original model.
(2) Introduction of an oblique fusion differential convolutional structure, which addresses the challenge of accurately identifying oblique edges in the presence of complex edge information. This novel structure improves upon previous methods that struggled with such oblique edge detection.
(3) We have incorporated a channel spatial attention module and an upsampling model, empowering the model to dynamically capture crucial information within each channel, suppress background noise, and extract image features across various scales and levels.

The remaining sections of the paper are organized as follows: Section 2 provides an overview of related research in the field of image edge detection. Section 3 presents, in detail, the proposed network architecture and the fusion difference convolution structure, along with their respective roles within the network. Section 4 describes the datasets used in the experiments, the evaluation metrics employed, and the experimental designs. Finally, Section 5 concludes the paper, summarizes the key findings, and suggests directions for future work.

## 2. Related Work

As a subtask of many advanced computer vision tasks, edge detection has been extensively studied in recent years. In the following sections, we will introduce the relevant research work that is related to our study.

Traditional Methods. Early edge detection algorithms were primarily based on carefully designed operators or filters using the local patterns of edges. They can be categorized into first-order operators, second-order operators, and others. Commonly used first-order operators include Roberts, Prewitt [28], Sobel [8], and Scharr. Commonly used second-order operators include Laplacian and LOG [10]. In addition, the Canny operator is a widely used edge detection operator, which remains highly effective in tasks with less stringent accuracy requirements. The principle behind these operators is to exploit the significant pixel transitions at image edges, where neighboring pixels exhibit large variations in their intensity values. In the operators, this manifests as the direction of the gradient that represents the maximum rate of grayscale change. These operators represent the earlier works, and modern developments have introduced numerous complex operators tailored for specific tasks. For example, the use of anisotropic Gaussian derivatives has been proposed to address corner detection. Another major category of traditional methods used for image segmentation is active contour models, such as the active contour-based image segmentation approach [29].

Biomimetic Approaches. Biomimetic edge detection methods are inspired by the visual systems of humans and other animals, aiming to simulate the perception and processing of edges in biological vision systems. These methods typically utilize image processing algorithms and computational models to mimic the functioning principles of biological neurons. Most biomimetic approaches aim to construct cortical cells, or nCRFs, to suppress noise and handle texture edges. For example, [30] improved the extraction of coarse contours and implemented texture suppression through multi-scale processing. Notably, some papers integrated nCRFs into deep learning methods for contour detection in natural images and achieved an ODS of 0.76 [31] on the BSDS500 dataset.

Deep Learning Methods. Due to certain limitations in terms of the speed and accuracy of traditional edge detection algorithms, deep-learning-based edge detection algorithms have gained significant attention in recent years. These algorithms leverage the characteristics of deep learning, such as multi-scale feature fusion, cascading mechanisms, and multi-task learning, to achieve higher accuracy and robustness. Based on the network architectures, the mainstream edge detection algorithms can be categorized into the following two types. The first type is based on the VGG16 network architecture, inspired by the fully convolutional network (FCN) [32]. Xie et al. introduced the pioneering end-to-end edge deep model called HED [13], which utilized side branches and fusion branches to compute losses and generate the final edge maps. Subsequent studies [33,34] made improvements to this model to obtain more accurate edge maps. The second type is based on the Transformer network architecture, which captures the complete image context and detailed local clues to extract meaningful and clear edge maps. An example of a commonly used model in this category is EDTER [18]. There are many research studies that utilize CNN models for edge detection. For instance, CNNs have been extensively employed in bridge rivet identification tasks, where they demonstrate remarkable performance [6] Both of the above-mentioned model types typically employ large backbone networks as pre-training models, which often results in high computational costs. To address this issue, some research works have made improvements. For instance, Pidinet [19] adopts an efficient backbone network and a given lateral structure for training, while LFFD [35] improves the relationship between effective receptive fields and facial features, proposing a fast face edge detection method.

## 3. Proposed Approach

This section introduces the specific network architecture. The overall network architecture in this paper is inspired by previous works [13,16,19,36]. We incorporate the four proposed fusion differential convolution (FDC) structures into the Xception network, replacing the separable convolutions in the original network architecture. Additionally, we introduce a channel spatial attention module (CSAM) before certain MaxPool layers to eliminate background noise. Finally, an up-sampling module (US) is introduced to avoid the loss of edge information that may occur as the network deepens, ultimately generating edge images.

In this section, we will introduce our network structure from three aspects: the overall neural network architecture, our newly proposed fusion difference convolution structure, and the loss function used in the network.

### 3.1. Neural Network Architecture

**Figure 2:**
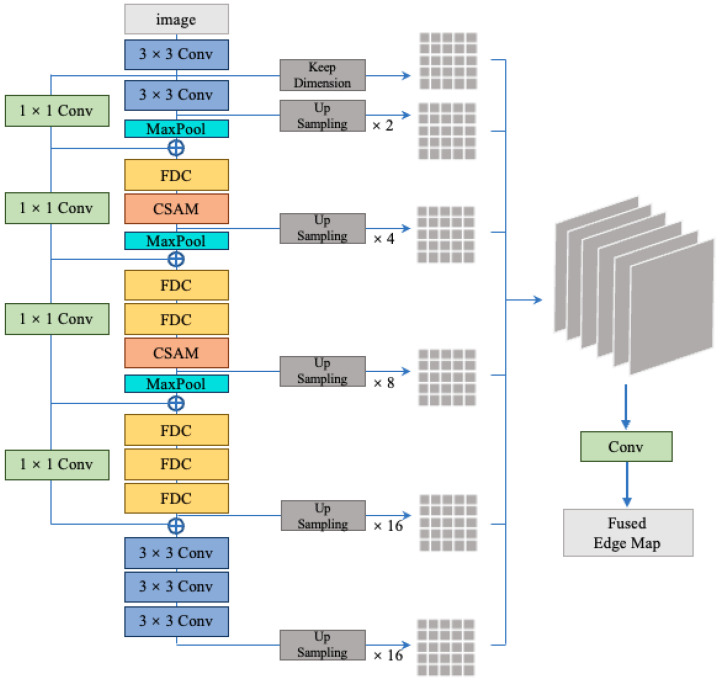
Proposed architecture. The backbone network is inspired by Xception, where the depth-wise separable convolution layers are replaced with regular convolutions and fusion difference convolution (FDC). The network incorporates the channel spatial attention module. The output of each main block is fed back to the upsampling block to generate intermediate edge maps, which are further combined to produce the fused edge map. More details are given in Section 3.

The network architecture is shown in Figure 2. Overall, the backbone network is inspired by the Xception network. Compared to the VGG16 backbone network, the Xception backbone network allows real-time inference on devices with limited resources. This advantage is due to the fact that VGG16 stacks more convolutional layers, resulting in a relatively larger number of parameters, which demands more computational resources and longer training times. Additionally, the VGG16 backbone heavily relies on well-initialized pre-trained weights. If trained entirely from scratch without loading any pre-trained weights, the performance of the actual edge detection images may not be ideal. On the other hand, the network based on the Xception architecture, with the improvements we made, can address this issue to a certain extent. The Xception backbone’s architecture allows for more efficient and effective feature extraction, which is particularly beneficial when training from scratch or with limited pre-training data. This improvement could lead to better edge detection performance even without heavy reliance on pre-trained weights.

However, unlike the original Xception network, we added an additional part at the end of the network with 256 filters, which does not include any max-pooling operations. While the depth-wise separable convolutions in Xception are more computationally efficient under resource constraints, we believe that regular convolutions have stronger expressive power when dealing with complex image features. Therefore, our model does not employ depthwise separable convolutions but instead commonly uses 3 × 3 convolutions. Additionally, in the subsequent subsection, we present a total of four fusion difference convolutional structures. We replace some of the regular convolutions with these structures. For more specific details, please refer to the next subsection. It is important to note that although we have four types of fusion convolutional structures, they are not used in parallel. Instead, only one type is chosen to replace each original convolution. We have extensively explored numerous combinations to optimize the model’s performance and attempted to provide the reasons behind the optimal combination. For detailed information, please refer to Section 4.

**Figure 3:**
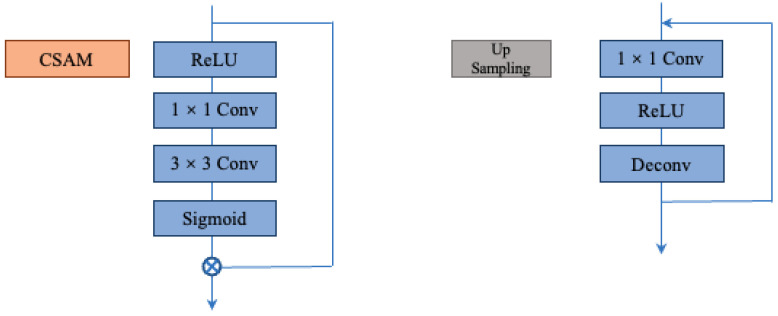
Details of the channel spatial attention module (CSAM) and the upsampling module.

In order to enable the network to adaptively capture more important information within channels and eliminate the influence of background noise, we introduced the channel spatial attention module (CSAM) before the MaxPool layer. The specific structure can be referred to in Figure 3. The steps are as follows: the M × H × W feature map is nonlinearly transformed using the ReLU activation function, and the results are sequentially fed into 1 × 1 and 3 × 3 convolutional layers for convolutional operations. This generates an attention weight map, which is then scaled by applying the Sigmoid activation function to obtain the attention weights. Finally, the attention weights are multiplied element-wise with the input feature map to obtain the final output. This structure encompasses reactions from all dimensions of the feature map, enabling the model to highlight important spatial positions while suppressing unimportant ones. This helps eliminate the texture details introduced by background noise, resulting in cleaner feature maps.

Additionally, we incorporated an up-sampling module in our network. We experimented with three up-sampling methods: deconvolution, bilinear interpolation, and unpooling. The experimental results showed that using deconvolution for up-sampling yielded the best performance. We believe this is because deconvolution preserves spatial information in the image. By learning trainable parameters, it adapts the up-sampling operation based on the characteristics of the dataset. Furthermore, we believe it can avoid the blurring or repeated textures introduced by interpolation methods. For specific experimental details, please refer to Section 4. The specific structure of our up-sampling module can be seen in Figure 3. It consists of a 1 × 1 convolutional layer, a ReLU function, and a deconvolution layer. The output channels of the deconvolution are the same as the input channels, and the specified kernel size and padding size are used. Each up-sampling operation doubles the spatial resolution. We repeat this module until the generated feature map matches the ground truth. This module achieves spatial expansion of the feature maps through up-sampling convolutional operations, allowing for up-sampling the low-resolution feature maps to the same size as the input image for pixel-level predictions.

Regarding skip connections, our model is similar to the one proposed by Xception [36]. We align the number of channels using a 1 × 1 convolution and directly add the input feature map to the output feature map, enabling the transfer of low-level detailed features to higher layers and facilitating faster propagation of bottom-level information to subsequent layers in the network. This structure enhances the generalization capability of our model, improving its perception and expressive power.

### 3.2. Fusion Difference Convolution

**Figure 4:**
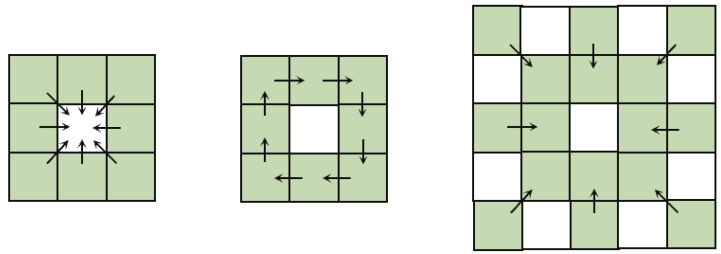
The difference convolution structure mentioned in Pidinet. These three structures integrate traditional edge detection operators into the popular convolutional operations in modern cellular neural networks, enhancing the ability to extract image gradient information and better obtaining image edge information.

Inspired by the improved local binary pattern difference (BIRD) [37] and the network architecture in Pidinet [19], we made modifications to the existing pixel difference convolution (PDC, Figure 4). First, we enhanced the three original PDCs by integrating vanilla convolutions. This new structure, referred to as fused difference convolution, was introduced to improve the original PDC by extracting more semantic information from the image, thereby reducing noise and texture information in the final edge image. Additionally, we proposed a novel type of difference convolution that strengthens the diagonal edge information in image features, resulting in a more accurate generation of diagonal edges compared to the original model.

In previous works, the extended local binary pattern (ELBP) has been proven effective in utilizing computed local pixel difference vectors to identify potential clues in images, complementing the visual task’s feature representation. Pidinet has already presented three types of pixel difference convolution structures. However, directly replacing regular convolutions with pixel difference convolutions leads to sensitivity to noise and loss of semantic information in shallow layers. Specifically, if a pixel is a noise point, the resulting pixel kernel within its eight-neighborhood during difference convolution is likely to be all 0 or all 1, rendering the original difference convolution structure ineffective. On the other hand, although the PDC structure directly extracts gradient information from the image, it tends to lose some of the original image’s semantic information in the shallow layers, resulting in missing edges in certain detailed regions of the final generated image.

**Figure 5:**
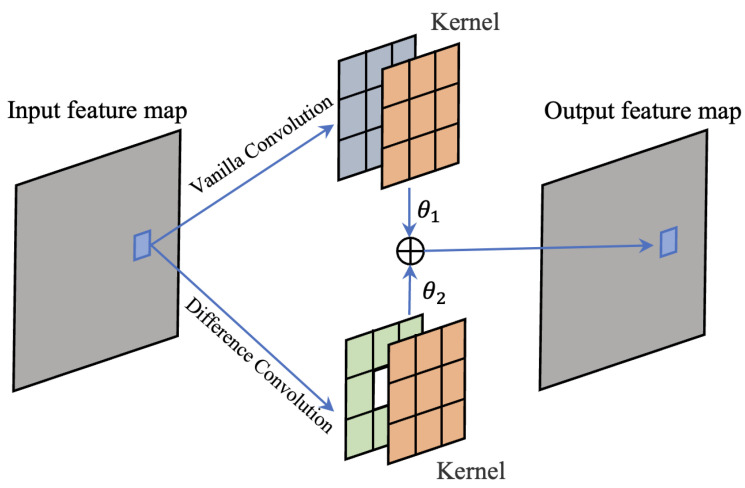
The fusion difference convolution (FDC). It introduces vanilla convolution into the original difference convolution layer and utilizes a hyperparameter theta to control the summation. This structure enables better extraction of both edge information and semantic information from the feature maps.

To address the aforementioned issues, we introduced vanilla convolution into the difference convolution structure, controlled by hyperparameters $\theta_{1}$ and $\theta_{2}$, both ranging from 0 to 1. These two convolutions share learnable weights. When $\theta_{1}=1$ and $\theta_{2}=0$, the structure degenerates into vanilla convolution, while $\theta_{1}=0$ and $\theta_{2}=1$ results in the PDC structure. This design allows us to tackle the two problems mentioned earlier. When a pixel is a noise point in the image, the addition of vanilla convolution enables us to learn meaningful image information. On the other hand, by relying on vanilla convolution, we can obtain the desired image semantic information even in the shallow layers of the network. The specific architecture can be seen in Figure 5.

**Figure 6:**
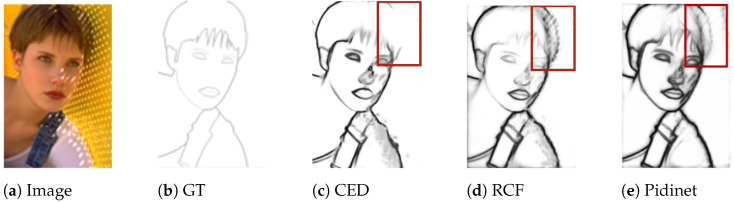
An example of using CED, RCF, and Pidinet methods can be observed. When facing partially slanted edges in an image, these methods may exhibit shortcomings such as missing edges or the inclusion of unwanted background information.

**Figure 7:**
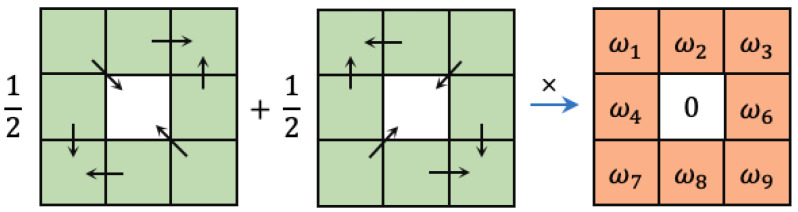
A new differential convolutional structure is proposed to capture slanted edge information in images.

On the other hand, we observed that in some existing network structures, such as CED [38], RCF, and Pidinet, the generated edge maps often lack diagonal edge information, or this information is blurred when dealing with complex texture or background information (see Figure 6). Therefore, we designed a new fusion difference convolution structure, incorporating the vanilla convolution as described earlier. This fusion difference convolution structure can be referred to in Figure 7. By subtracting adjacent elements along the main and off-diagonal, this structure enhances the gradient information for diagonal edges, enriching the diagonal edge information while suppressing the surrounding texture and background noise. From the experimental results, it is evident that the inclusion of this structure effectively improves the ability to extract diagonal edge information from images.

In addition, following the approach of [19], we transformed the aforementioned four fusion difference convolutions into vanilla convolutions. This transformation significantly reduces the computational cost and memory footprint, allowing our model to perform inference operations with almost the same computational complexity as the original vanilla convolution structure. Taking one of the fusion difference convolution structures depicted in Figure 7 as an example, we applied the following equation for the conversion:

(1) $$\begin{array}{cc} y & =\theta_{1}\sum_{i}x_{i}w_{i}+\theta_{2}\left[\frac{1}{2}\left(2x_{1}-x_{5}\right)w_{1}+\frac{1}{2}\left(2x_{2}-x_{1}-x_{3}\right)w_{2}+\cdots \right]\\ \; & =\left[\theta_{1}w_{1}+\frac{\theta_{2}}{2}\left[2w_{1}-w_{2}-w_{4}\right]\right]x_{1}+\left[\theta_{1}w_{1}+\theta_{2}w_{2}\right]x_{2}+\cdots \\ \; & ={\hat{w}}_{1}x_{1}+{\hat{w}}_{2}x_{2}+\cdots =\sum_{i}{\hat{w}}_{i}x_{i} \end{array}$$

The conversion formulas corresponding to the other three FDCs can be referred to in Appendix A. By applying this transformation, we achieve comparable performance while reducing the computational overhead associated with the fusion difference convolutions.

### 3.3. Loss Function

We adopt a weighted cross-entropy loss function similar to HED [13] for our network. The use of a weighted cross-entropy loss function confers distinct advantages in addressing class imbalance, particularly in edge detection tasks where class imbalances prevail. In most cases, there are significantly fewer edge pixels than non-edge pixels in an image, leading to an imbalanced class distribution. Therefore, employing the weighted cross-entropy loss enables the model to focus more on the edge class, thereby enhancing edge detection performance. Additionally, the weighting mechanism in the loss function balances the gradients during training by treating different classes differently. As a result, the model can converge more efficiently and achieve superior results.

We denote our network training set as:

(2) $$S=\left\{\left(X_{n},Y_{n}\right),n=1,\ldots ,N\right\}$$

where $X_{n}$ represents the preprocessed images, and $Y_{n}$ represents the binary edge labels of $X_{n}$. Let W denote all the parameters of the network, and we define the parameters of each side branch as:

(3) $$w=\left(w^{\left(1\right)},\ldots ,w^{\left(M\right)}\right)$$

The loss function can be defined as follows:

(4) $$L\left(W,w\right)=\sum_{m=1}^{M}\alpha_{m}\ell^{\left(m\right)}\left(W,w^{\left(m\right)}\right)$$

where $\alpha_{m}$ represents the weights of each side branches’ loss function. Additionally, $\ell^{\left(m\right)}$ denotes the loss function for each side branch, specifically the cross-entropy loss function, which is defined as follows:

(5) $$\begin{array}{c} \ell^{\left(m\right)}\left(W,w^{\left(m\right)}\right)=-\beta \sum_{j\in Y_{+}}\log\mathrm{Pr}\left(y_{j}=1|X;W,w^{\left(m\right)}\right)\\ -\left(1-\beta \right)\sum_{j\in Y}\log\mathrm{Pr}\left(y_{j}=0|X;W,w^{\left(m\right)}\right) \end{array}$$

where β represents the class balance weights for both positive and negative samples in edge detection.

(6) $$\beta =\left|Y^{-}\right|/\left|Y^{+}\left|+\right|Y^{-}\right|$$

(7) $$\left(1-\beta \right)=\left|Y^{+}\right|/\left|Y^{+}\left|+\right|Y^{-}\right|$$

where $|Y^{-}|$ and $|Y^{+}|$ denote the edge and non-edge in the ground truth. Additionally, $\mathrm{Pr}\left(y_{j}|X;W,w^{\left(m\right)}\right)$ represents the predicted edge values for the m-th side branch, which have been mapped to (0,1) using the sigmoid activation function.

## 4. Experiments

### 4.1. Datasets

The main training dataset used in this paper is the BIPED dataset. It consists of 258 street images, each with a high-definition resolution of 1280 × 720 pixels. These images have been carefully processed and meticulously annotated by experts in the field of computer vision. Furthermore, all results have undergone cross-validation, ensuring a low possibility of erroneous or missing edges. The BIPED dataset is a publicly available dataset and serves as a benchmark for evaluating our edge detection algorithm.

It is worth noting that many previous edge detection papers primarily utilized datasets such as BSDS, MDBD, NYUD, and PASCAL. Among these datasets, only MDBD is specifically designed for edge detection tasks. However, the annotations in the MDBD dataset suffer from missing and erroneous edges, which can penalize the edge detection network during the training process. Therefore, in recent years, many papers have chosen to train and validate their models using the BIPED dataset.

To assess the performance of our model, we conducted testing on the BIPED test set. Additionally, to provide a comprehensive evaluation of the model’s performance, we also conducted testing on four other datasets: BSDS, MDBD, NYUD, and PASCAL. It is important to note that our model was not trained on these four datasets directly. Instead, we used the pre-trained weights from the BIPED network for validation purposes. In contrast, other methods have undergone training on at least one of the aforementioned four datasets. Next, we will introduce the above four datasets in detail.

BSDS [24]. The Berkeley Segmentation Dataset is primarily used for image segmentation and boundary detection. It has two versions, BSDS300 and BSDS500. Currently, the majority of edge detection networks are trained and validated using the images from BSDS300, and testing is performed using an additional 200 images. Each image in the BSDS dataset is annotated by 3-6 annotators, and therefore, to obtain ground truth for the images, an overlay operation is performed before training.

MDBD [25]. The Multicue Dataset for Boundary Detection is primarily used for edge detection tasks. It comprises 100 high-definition images (1280 × 720) with meticulous annotations. Typically, 80 randomly selected images are used as the training set, while the remaining 20 images serve as the test set. In our work, we evaluated our model on a randomly chosen subset of 20 images from this dataset.

NYUD [26]. The New York University Dataset is commonly used, with NYUD V2 being the preferred version. It contains 1449 images of size 640 × 480. Usually, 654 images are selected as the training set, while the remaining images are used for testing and validation. The dataset mainly consists of indoor scene data, which differs significantly from outdoor scenes in the BIPED dataset. Therefore, our model did not achieve higher metrics than previous networks in terms of ODS, OIS, and other evaluation measures.

PASCAL [27]. The Pascal Context dataset is primarily used for semantic image segmentation tasks. The most commonly used version is PASCAL VOC 2012, which includes 11,530 images. This dataset only provides the true contour value of the image, so its evaluation index for edge detection tasks will be very low. We selected 500 images from this dataset for testing our network.

### 4.2. Implementation Details

We randomly selected 50 images from the BIPED dataset for the test set, while the remaining 208 images were used for the training set. Prior to training, data augmentation was applied to these 208 images, which were originally sized at 1280 × 720. Each image underwent rotation at various angles and was cropped using an internal square to obtain a final size of 512 × 512. Additionally, the images underwent two rounds of gamma correction (0.3030 and 0.6060). This data augmentation process generated 100 new images for each original image, resulting in a total of 20,800 images in our training set.

**Figure 8:**
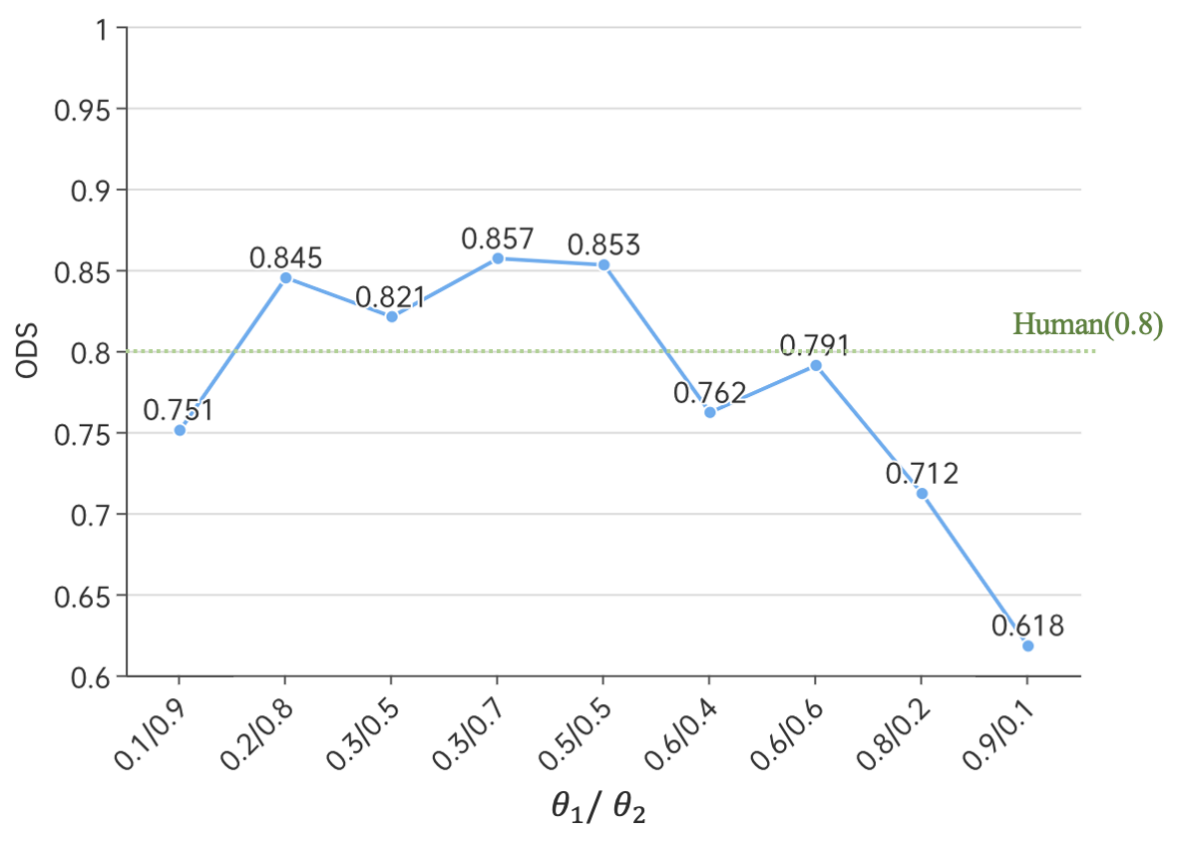
Line chart of super parameter $\theta_{1},\theta_{2}$, and ODS. The X-axis represents hyperparameters $\theta_{1}$ and $\theta_{2}$, and the Y-axis represents the output image ODS value. It can be seen that when $\theta_{1}=0.3$ and $\theta_{2}=0.7$, the model reaches its optimal state.

To obtain the best edge detection model, we experimented with various combinations of hyperparameters $\theta_{1}$ and $\theta_{2}$. We achieved the optimal solution (ODS 0.857) when setting $\theta_{1}=0.3$ and $\theta_{2}=0.7$. Please refer to Figure 8 for the corresponding curves. We believe that this can be explained by the fact that in our model, a higher weight is assigned to the FDC component. This difference convolution is better at extracting gradient information from the images, which primarily reflects the edge information. This weighting allows the model’s output to align more closely with the ground-truth edge information. On the other hand, vanilla convolutions are more focused on capturing semantic information in the images, helping to eliminate unnecessary textures and background noise. Therefore, the weight assigned to this component is slightly lower.

The network model was implemented using PyTorch, with hyperparameters set as $\theta_{1}=0.3$ and $\theta_{2}=0.7$. The initial learning rate was set to 1 × 10^−4^, and the training was conducted for 17 epochs with a batch size of 16. All experiments were performed on an RTX 3090 GPU. The training time for the network was approximately 7.3 h. During inference, the model achieved a processing speed of 28.8 fps on an RTX 3090 GPU.

The evaluation of edge detection has been well-defined in previous works. In this paper, we consider widely used evaluation metrics, including optimal dataset scale (ODS), optimal image scale (OIS), and average precision (AP). It should be pointed out that unnecessary background information in the final generated edge image can to some extent affect the above three evaluation indicators. Therefore, we provide the definitions of the three indicators and the impact of background information here.

ODS: ODS evaluates the performance of an edge detection algorithm at different threshold levels. It considers the numbers of true positives (correctly detected edge pixels), false positives (incorrectly detected edge pixels), and true negatives (undetected edge pixels) at different thresholds, calculating a comprehensive detection rate. Background details can affect the ODS metric because complex or cluttered backgrounds may lead to more false positives or obscure genuine edges, thus reducing the ODS score. OIS: OIS is a variation of ODS, aiming to find an optimal threshold that maximizes edge detection performance. OIS takes into account the performance under different image scales and selects the best threshold accordingly. Background details can also influence the OIS metric because the complexity of the background may affect the saliency of edges at different scales, impacting the choice of the optimal threshold.

AP: AP is a metric used to evaluate object detection and edge detection tasks. It primarily assesses the balance between precision and recall at different thresholds. Background details can similarly affect the AP metric as complex backgrounds may lead to inaccurate edge localization, reducing the algorithm’s precision.

In edge detection models, precision refers to the probability that the machine-generated boundary pixels in edge detection are true boundary pixels. Recall, on the other hand, represents the probability of detecting true boundary pixels in edge detection out of all the true boundary pixels.

(8) $$\begin{array}{c} \;\mathrm{Precision}\;=\frac{TP}{TP+FP} \end{array}$$

(9) $$\begin{array}{c} \;\mathrm{Recall}\;=\frac{TP}{TP+FN} \end{array}$$

where *TP* stands for true positive, *FP* stands for false positive, and *FN* stands for false negative. ODS refers to selecting a fixed threshold value, denoted as η, that maximizes the F-score on the dataset. OIS, on the other hand, involves selecting the threshold value, denoted as η, for each image individually to maximize its F-score.

### 4.3. Results

To achieve the best performance of our model, we conducted extensive experiments on two modules: FDC and US. First, we discuss the experiments on the FDC module. It should be noted that we have a total of four FDC variants and one vanilla convolution, making a total of five convolution layers to replace the original convolution layers in Xception. However, trying all possible combinations to find the optimal solution is clearly impractical. Therefore, we only explored a subset of combinations and attempted to provide insights into the potential optimal solution of the model. Experimental data can be referred to in Table 1. It can be observed that replacing all convolution layers with our FDC module does not improve the model’s performance. Instead, it performs worse than using vanilla convolutions alone. We speculate that this may be due to two reasons. Firstly, the extensive use of FDC modules may only effectively extract gradient features from the images. Although we incorporate vanilla convolutions into the FDC module, as the network goes deeper, the extracted image features become increasingly blurry, thus neglecting other crucial image features. Secondly, shallow layers of the network require vanilla convolutions to rapidly acquire semantic information from the images and pass it to the subsequent layers. By directly using the FDC module, the network might inadvertently discard some useful feature information in the shallow layers, making it challenging to recover this information.

The optimal configuration found to be most effective is [C,A]×2-[R,D]×1. This means that each type of FDC is used in the model, and they will extract gradient information from different encoding directions in the image. This diverse incorporation of FDC types enriches the model’s feature information, leading to the generation of more accurate edge images. By employing a combination of different FDC types, our model benefits from a broader range of gradient information, which improves its ability to produce more precise edge detection results. This finding supports the effectiveness of the selected configuration and reinforces the significance of employing a variety of FDC types in the model architecture.

Moving on to the consideration of the up-sampling (US) module, we experimented with three approaches: deconvolution, bilinear interpolation, and unpooling, as discussed in detail in Section 3. The experimental results can be found in Table 2. Overall, we observed that the differences between these three up-sampling methods were not significant. However, using deconvolution for up-sampling yielded slightly better results compared to the other two methods. Therefore, in the subsequent discussions of the experimental results, we validate the model using the best-performing configuration as indicated by the aforementioned metrics.

**Figure 9:**
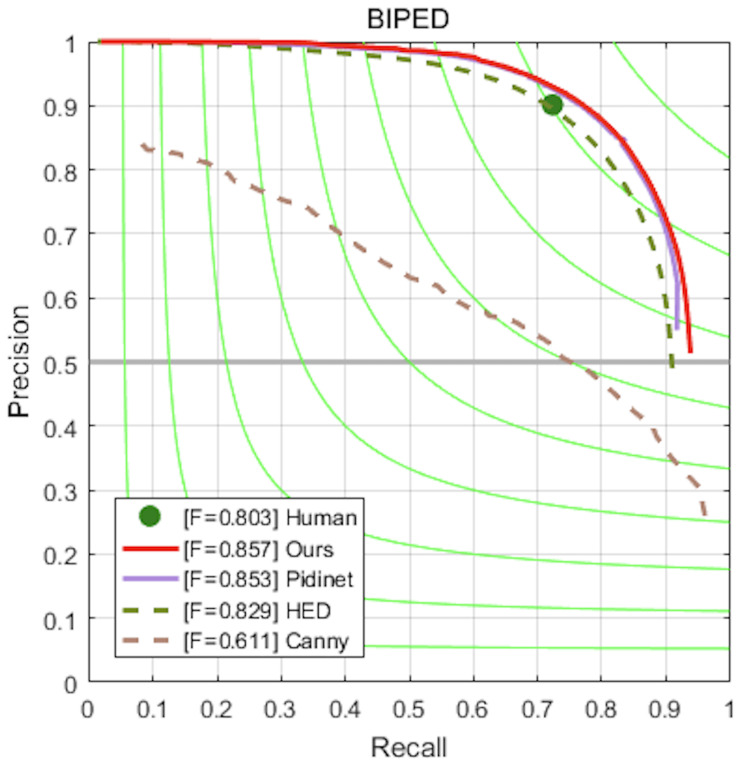
Precision–recall curves of our models and some competitors on BIPED dataset.

We conducted training on the BIPED dataset, and it is worth noting that our model was trained completely from scratch without utilizing pre-trained weights from backbone networks. We have provided relevant data for each output, as shown in Table 3. The experimental results are presented in Table 4. In comparison to HED, RCF, BDCN, and Pidinet, which incorporate pre-trained weights from backbone networks, our approach outperforms them in terms of performance. Specifically, we achieved a performance of 0.829 vs. 0.857 in ODS and 0.841 vs. 0.854 in OIS for our method compared to HED. Although our method slightly lags behind Pidinet in terms of the OIS metric, we still achieved a 0.4% improvement in ODS, and a 0.5% improvement in AP. At the same time, compared to our baseline (HED), we achieved a 3.3% improvement in ODS, a 1.5% improvement in OIS, and a 3.2% improvement in AP. In order to provide a more intuitive evaluation of our model, we have drawn recall precision, as shown in Figure 9.

**Figure 10:**
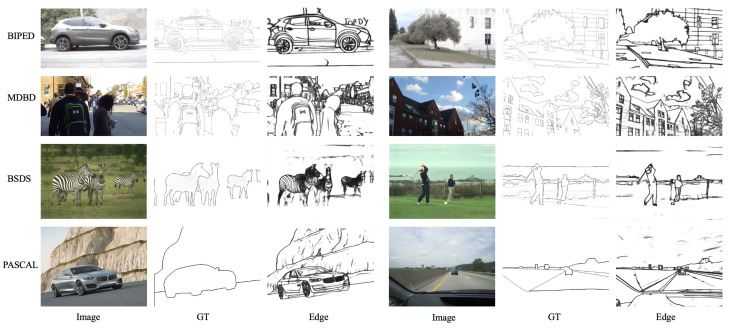
Results from the proposed approach. We have provided two examples for each dataset. We can see that our edge image results show good overall and detailed performance on BIPED dataset. Since we did not train on MDBD, BSDS, and PASCAL, they will not extract accurate edges from all images on the corresponding test set. We have only selected some well performing images for display.

To provide a fairer comparison with previous methods, we conducted validation on four datasets: MDBD, BSDS500, NYUD, and PASCAL. The detailed results can be found in Table 5 and Figure 10. We categorized the datasets into two types: edge detection datasets and contour/boundary detection/segmentation datasets. It can be observed that our method achieved comparable results to previous methods only on the MDBD dataset but fell short of ideal results on the other datasets. There are several reasons for this. The main reason is that our model was not trained on these datasets but rather validated using the model trained on the BIPED dataset. In contrast, other methods were trained at least once on the respective datasets and may have undergone extensive fine-tuning experiments. Additionally, the MDBD dataset is the only carefully annotated edge dataset among the four, and its images share more similarities with the BIPED dataset in terms of scenes. Therefore, the weights of our trained model may be more biased toward recognizing edges in similar scenes. Lastly, the other datasets are not specifically designed for edge detection, which means their evaluation metrics penalize the edge images obtained by our model. Hence, we reasonably speculate that when we carefully annotate images from the other datasets, our model will achieve similar or even higher performance compared to previous models.

### 4.4. Ablation Study

To demonstrate the effectiveness of our model and identify the optimal model architecture, we conducted ablation experiments on the BIPED dataset. In Table 1 and Table 2, we provided potential optimal solutions for the up-sampling module (using deconvolution) and FDC module, respectively. It can be observed that both modules contribute to the performance improvement of our model, as indicated by the results.

In this section, we focused on conducting ablation experiments on three modules: FDC, CSAM, and US. The experimental results can be found in Table 6. It is evident that all three modules lead to performance improvements in our model. We noticed that removing the US module from the network did not result in a significant drop in ODS/OIS values. This may be attributed to the fact that the skip connections in the network have already conveyed some shallow-level image features to the higher layers. However, incorporating the US module still enhances the performance of our model, indicating its value. On the other hand, we observed that the inclusion of the CSAM module does not cause a significant decrease in ODS/OIS values compared to its exclusion. Hence, when dealing with edge detection tasks that have lower accuracy requirements, it is advisable to consider removing the CSAM attention module to lighten the network.

## 5. Conclusions

In conclusion, our contributions can be summarized in three parts.

1. We proposed four novel fusion difference convolution (FDC) structures. Among them, we introduced a new differential convolution to address the weakness of previous methods in recognizing complex oblique edges. These four FDC structures, by combining differential convolutions with vanilla convolutions, achieved more accurate and robust edge detection.

2. We presented an improved model based on Xception. By incorporating the FDC, CSAM, and US modules, our model was able to be trained from scratch with limited data and ultimately achieved performance surpassing human-level accuracy. This breakthrough deviated from the conventional practice of relying on pre-trained backbone network weights, such as VGG16, in edge detection networks.

3. We conducted training and validation on the carefully annotated BIPED dataset, as well as extensive edge detection experiments on the MDBD, BSDS500, NYUD, and PASCAL datasets. Our experimental results demonstrated that our network architecture achieved promising performance. Overall, our contributions lie in the development of novel FDC structures, the improvement of the Xception-based model with FDC, CSAM, and US modules, and the thorough evaluation of our approach on multiple datasets.

We envision the application of our model in practical industrial production to replace some manual labor. Additionally, as mentioned in Section 1, edge detection is a subtask of many advanced visual tasks, such as semantic segmentation or object detection. We aim for our model to be applied in these related tasks as well. By leveraging the accurate and stable edge detection capabilities of our model, we believe we can contribute to improving the performance and efficiency of various computer vision applications.

## Figures and Tables

**Table 1:** Possible configurations of our method. ‘C’, ‘A’, ‘R’, ‘D’, and ‘V’, respectively, represent the four FDC modules and vanilla convolutions we provide. We have a total of 11 convolutional layers that can be replaced. If there is no special indication, the first 2 and last 3 layers of the network are vanilla convolutions. Therefore, the replacement mentioned above is hidden for less than 11 convolutional layers in the table.

**Architecture**	V×11	C×11	A×11
**ODS/OIS**	0.802/0.811	0.711/0.721	0.678/0.624
**Architecture**	C×7	C×5-D×2	R×5-D×2
**ODS/OIS**	0.817/0.819	0.818/0.821	0.744/0.748
**Architecture**	C×4-[A,R,D]×1	D×4-[C,A,R]×1	[A,R]×2-[C,D]×1
**ODS/OIS**	0.855/0.850	0.813/0.824	0.719/0.732
**Architecture**	[C,D]×2-[A,R]×1	baseline	[C,A]×2-[R,D]×1
**ODS/OIS**	0.850/0.848	0.829/0.841	**0.857/0.854**

**Table 2:** Evaluation indicators for different up-sampling methods where US-rp means that the anti-pooling operation is used to realize the up-sampling, US-b means that the Bilinear interpolation is used to realize the up-sampling, and US-dc means that the deconvolution is used to realize the up-sampling.

US Method	ODS	OIS	AP
US-rp	0.849	0.852	0.892
US-b	0.855	0.851	0.890
US-dc	**0.857**	**0.854**	**0.897**

**Table 3:** Quantitative evaluation of the 7 predictions of our method on BIPED test dataset.

Outputs	ODS	OIS	AP
Outputs 1(${\hat{y}}_{1}$)	0.722	0.751	0.718
Outputs 2(${\hat{y}}_{2}$)	0.738	0.779	0.781
Outputs 3(${\hat{y}}_{3}$)	0.827	0.799	0.843
Outputs 4(${\hat{y}}_{4}$)	0.846	0.836	0.859
Outputs 5(${\hat{y}}_{5}$)	0.851	0.838	0.869
Outputs 6(${\hat{y}}_{6}$)	0.854	0.841	0.892
Edge Map	0.857	0.854	0.897

**Table 4:** Train with BIPED dataset and compare the evaluation indicators obtained between methods.

Methods	ODS	OIS	AP
HED [13]	0.829	0.841	0.869
RCF [39]	0.841	0.859	0.882
BDCN [40]	0.839	0.853	0.887
Pidinet [19]	0.853	0.860	0.893
Ours	0.857	0.854	0.897

**Table 5:** The performance of the network in this article on other datasets (values from other approaches come from the corresponding publications).

Dataset	Methods	ODS	OIS	AP
**Edge Detection Dataset**				
MDBD [25]	HED [13]	0.841	0.864	0.887
	RCF [39]	0.851	0.862	-
	BDCN [40]	0.855	0.856	0.887
	Pidinet [19]	0.845	0.861	0.890
	Ours	0.857	0.851	0.893
**Contour/Boundary Detection/Segmentation Datasets**				
BSDS500 [24]	HED [13]	0.788	0.808	0.811
	RCF [39]	0.806	0.823	-
	BDCN [40]	0.82	0.838	0.840
	Pidinet [19]	0.807	0.823	0.832
	Ours	0.730	0.778	0.747
NYUD [26]	HED [13]	0.720	0.761	0.786
	RCF [39]	0.743	0.757	-
	BDCN [40]	0.749	0.751	0.781
	Pidinet [19]	0.741	0.744	0.778
	Ours	0.602	0.615	0.490
PASCAL [27]	CED [38]	0.726	0.750	0.778
	HED [13]	0.514	0.542	0.389
	RCF [39]	0.501	0.526	-
	Ours	0.515	0.539	0.385

**Table 6:** The results of the ablation experiment. The models are trained with BIPED training set and evaluated on BIPED [16].

FDC	CSAM	US	ODS/OIS
✕	✓	✓	0.802/0.811
✕	✕	✓	0.792/0.804
✓	✕	✓	0.841/0.836
✓	✓	✕	0.839/0.835
✓	✓	✓	0.857/0.854

## Data Availability

Not applicable.

## Abbreviations

The following abbreviations are used in this manuscript:

AP	Average Precision
CNN	Convolutional Neural Network
CSAM	Channel Spatial Attention Module
FDC	Fusion Difference Convolution
FN	False Negative
FP	False Positive
GT	Ground Truth
ODS	Optimal Dataset Scale
OIS	Optimal Image Scale
PDC	Pixel Difference Convolution
ReLU	Rectified Linear Unit
TP	True Positive
US	Up-Sampling Module

## Appendix A

In Section 3, we have provided a formula for converting FDC to vanilla convolution. Here, we provide the remaining three formulas for converting FDC to vanilla convolution.

(A1) $$\begin{array}{cc} y & =\theta_{1}\sum_{i}x_{i}w_{i}+\theta_{2}\cdot \sum_{i}\left(x_{i}-x_{5}\right)w_{i}\\ \; & =\left(\theta_{1}w_{1}+\theta_{2}w_{1}\right)x_{1}+\left(\theta_{1}w_{2}+\theta_{2}w_{2}\right)x_{2}+\cdots \\ \; & ={\hat{w}}_{1}x_{1}+{\hat{w}}_{2}x_{2}+\ldots =\sum_{i}{\hat{w}}_{i}x_{i} \end{array}$$

(A2) $$\begin{array}{cc} y & =\theta_{1}\sum_{i}x_{i}w_{i}+\theta_{2}\left[\left(x_{1}-x_{2}\right)w_{1}+\left(x_{2}-x_{3}\right)w_{2}+\cdots \right]\\ \; & =\left[\theta_{1}w_{1}+\theta_{2}\left(w_{1}-w_{4}\right)\right]x_{1}+\left[\theta_{1}w_{2}+\theta_{2}\left(w_{2}-w_{1}\right)\right]x_{2}+\cdots \\ \; & ={\hat{w}}_{1}x_{1}+{\hat{w}}_{2}x_{2}+\ldots =\sum_{i}{\hat{w}}_{i}x_{i} \end{array}$$

(A3) $$\begin{array}{cc} y & =\theta_{1}\sum_{i}x_{i}w_{i}+\theta_{2}\left[\left(x_{1}-x_{7}\right)w_{1}+\left(x_{3}-x_{8}\right)w_{2}+\cdots \right]\\ \; & =\left(\theta_{1}w_{1}+\theta_{2}w_{1}\right)x_{1}+\left(\theta_{1}w_{2}+\theta_{2}w_{2}\right)x_{3}+\ldots \\ \; & ={\hat{w}}_{1}x_{1}+{\hat{w}}_{2}x_{2}+\ldots =\sum_{i}{\hat{w}}_{i}x_{i} \end{array}$$
